# Synergistic enhancement of magnetic, dielectric, and optical properties in vanadium-substituted LaMnO_3_ multiferroics

**DOI:** 10.1039/d6ra04264c

**Published:** 2026-11-02

**Authors:** Mohammed Ahmed Wahba, Rabab K. Khaled

**Affiliations:** a Department of Inorganic Chemistry, National Research Centre 33 El Buhouth St. (former Eltahrir St.), Dokki Giza 12622 Egypt mohamedwahba12@gmail.com; b Department of Physical Chemistry, National Research Centre 33 El Buhouth St. (former Eltahrir St.), Dokki Giza Egypt rababkhaled39@yahoo.com

## Abstract

Perovskite manganites, specifically LaMnO_3_, emerge as promising candidates for multiferroic and multifunctional devices arising from charge, spin, lattice coupling interplay. Herein, we present B-site vanadium substitution, La(Mn_1−*x*_V_*x*_)O_3_ (*x* = 0–0.05, sol–gel synthesized) as an effective route for tuning these properties. XRD demonstrates dominant trigonal *R*3̄*c* phase with minor V_2_O_3_/La_2_O_3_ secondary phases at V-loading above 1%, while unit cell volume shows sharp expansion at low doping levels before saturating. V-substitution induces a progressive blue shift in the absorption edge, with band gap increases (from 0.768 to 0.886 eV) *via* bandwidth narrowing and a Burstein–Moss-type effect, and drives a 13-fold enhancement in dielectric constant through Maxwell–Wagner–Sillars interfacial polarization. Magnetically an enhancement in room-temperature ferromagnetism was attained with saturation magnetization reaching 3.52 emu g^−1^ (LMV5) and coercivity peaking at LMV3 (140% increase over the pristine LaMnO_3_). These results establish vanadium as a versatile dopant for engineering multifunctional manganites.

## Abstract 2

B-site vanadium substitution, La(Mn_1−*x*_V_*x*_)O_3_ (*x* = 0–0.05, sol–gel synthesized) is presented as an effective route for tailoring the structural, optical, electrical, and magnetic properties of LaMnO_3_.

## Graphical abstract

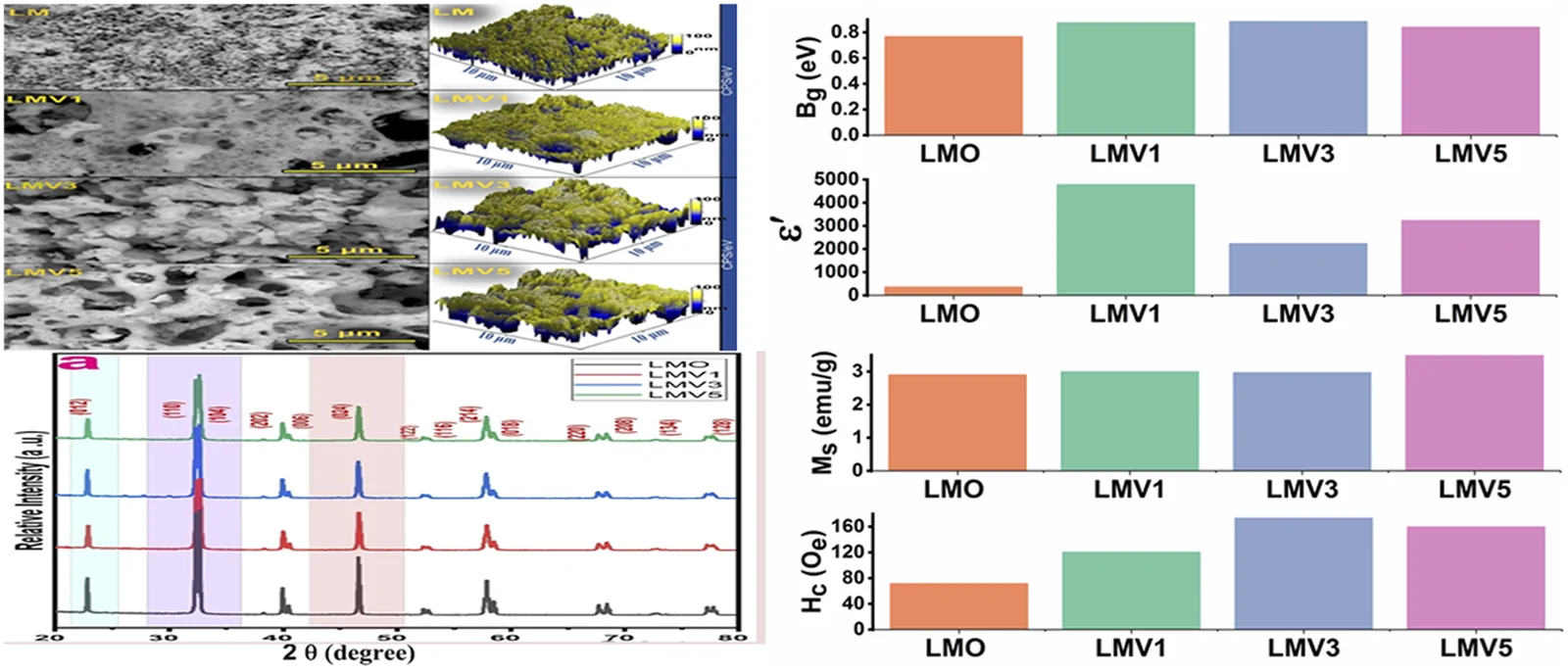

## 1. Introduction

Multiferroic perovskite lanthanum manganite, LaMnO_3_ (LMO), have attracted substantial research interest owing to the rich interplay of their structural, electronic, optical, and magnetic phenomena.^1–3^ LaMnO_3_ crystallizes in numerous polymorphs including orthorhombic, rhombohedral, cubic, or trigonal and hexagonal phases depending on temperature and oxygen-stoichiometry.^4,5^ In the perovskite structure of LaMnO_3_, the corner-sharing MnO_6_ octahedra form a three-dimensional framework, while La^3+^ cations occupy the larger A-site cavities within the octahedral network. The mixed-valence nature of manganese, Jahn–Teller distortions, orbital ordering, and strong electron-lattice coupling, collectively generate functional behaviors valuable for photocatalysis, spintronics, ceramic fuel cells, and magneto-optical apparatus.^6–8^ The delicate balance of Mn^3+^/Mn^4+^ ratio, oxygen stoichiometry, and framework distortions play a decisive role that governs the magnetic interactions, electronic structure, and dielectric response.

Bulk magnetism of LaMnO_3_ is sensitive to stoichiometry and crystal structure. Stoichiometric orthorhombic LaMnO_3_ (*Pnma*) is A-type antiferromagnet with *T*_N_ ≈ 140 K and a small Dzyaloshinskii–Moriya-canted ferromagnetic moment.^9^ Oxygen excess or cation deficiency LaMnO_3+*δ*_, which typically has a rhombohedral (*R*3̄*c*) structure, has mixed Mn^3+^/Mn^4+^ and can be ferromagnetically ordered by double-exchange, with Curie temperatures reported to range from 135–240 K depending on the degree of non-stoichiometry.^10^ Enhancement of LaMnO_3_ multifunctional properties have historically focused on A-site substitution strategies with divalent alkaline-earth cations such as Ca^2+^, Sr^2+^, and Ba^2+^.^11–13^ This substitution introduces Mn^4+^ species through charge-compensation processes (2Mn^3+^ → Mn^2+^ + Mn^4+^ or Mn^3+^ → Mn^4+^ + e^−^). The Mn^3+^/Mn^4+^ mixed-valence state dramatically alters the electronic bandwidth, lattice distortion, and magnetic interactions. The enhanced Mn^3+^/Mn^4+^ electron hopping promotes the well-known double-exchange mechanism, strengthening ferromagnetic ordering while simultaneously influencing electrical conductivity, metal–insulator transitions, and colossal magnetoresistance (CMR).^8,14^ As a result, compositions such as La_0.7_Ca_0.03_MnO_3_ exhibited significant structural modifications with reduced orthorhombic distortion compared to pristine LaMnO_3_, which resulted in enhanced electrical conductivity, and colossal magnetoresistance effects. Furthermore, the mixed-valence state enhanced dielectric constants by two orders of magnitude and optimized the optical bandgap through adapting interfacial polarization, space charge effects, electron mobility.^11,15^ Similarly, incorporation of Na, Sr, Dy, Er, and Ce dopants in LaMnO_3_ at 5% (La_0.95_X_0.05_MnO_3_) modifies the Mn–O–Mn bond geometry and carrier concentration, enhancing double-exchange interactions and the Curie temperature and electrical conductivity compared to the unsubstituted material. The dopant-type and concentration further tune magnetic anisotropy, coercivity, and metal-insulator transitions, which enables controlled tailoring of structural, magnetic, and magneto-transport properties.^16^ It is also reported that substitution of Ba-doped LaMnO_3_ enhances optical absorptivity and improves electronic transitions. Across a broad wavelength range by increasing free carrier concentration, reducing bandgap energy, and introducing higher oxygen content.^17^ These compositional variations demonstrate the valuable influence of A-site substitution on simultaneously modulation of structural, electronic, optical, and functional properties.

Alternatively, substitution at the Mn-position (B-site) provides a fundamentally different and highly effective pathway for tailoring the structural, electronic, and magnetic performance of LaMnO_3_. Replacement of Mn with transition-metal dopants such as Fe^3+^, Co^3+^, Ni^2+^, Cu^2+^, or Rh^3+^, directly adjusts the Mn–O–Mn super exchange network, introduces new d-electron configurations that alters octahedral distortions, charge-compensation mechanisms, and orbital ordering tendencies. Such substitutions can result in rich phase competition involving ferromagnetic, antiferromagnetic, or spin-glass states. It can also influence Jahn–Teller distortions, and affect transport mechanisms through changes in carrier density and bandwidth. For instances, the catalytic efficiency of LaMnO_3_ was enhanced by Rh-doping through synergistic redox activity for simultaneous NO reduction and CO/hydrocarbon oxidation. Cobalt doping LaMnO_3_ was also reported to promote charge-transfer through generation of varying oxidation states thus improving oxygen evolution reaction (OER) electrocatalytic performance.^18^ A significant enhancement in optical and visible light photocatalytic activity was also achieved through doping of LaMnO_3_ with Fe, Fe/Co, and Fe/Ni *via* improving separation of charge carriers.^2^ Additionally, Cu-incorporation in LaCu_*x*_Mn_1−*x*_O_3_ led to magnetism tuning from ferromagnetic to antiferromagnetic.^19^ Comparative studies on A-site and B-site double-doped systems such as La_0.8_Sr_0.1_Ca_0.1_MnO_3_ and LaMn_0.8_Fe_0.1_Co_0.1_O_3_ further demonstrated that optimization of doping modifies structural symmetry and magnetic hardness which enables precise magnetic transitions suitable for advanced device applications.^20^ These findings highlight that B-site doping offers a versatile strategy for engineering multifunctional properties of LaMnO_3_.

Although the widely examined B-site doping of LaMnO_3_ with 3d transition metals, vanadium substitution in LaMnO_3_ remains far less explored than other 3d transition-metal B-site dopants. Herein, V-incorporation influence on the structural, optical, electrical, and magnetic behavior of LaMnO_3_ was investigate. V-substitution are promising approach due to the variation in oxidation state, charge-compensation pathways, and the resulting structure–property effects. Vanadium can adopt multiple oxidation states (V^2+^–V^5+^) with markedly different ionic radii and d-electron configurations. The incorporation of vanadium in LaMnO_3_ lattice is expected to generate distinct defect chemistries that strongly influence transport, dielectric polarization, optical absorption, and magnetic interactions. The V^5+^, Mn^3+^, Mn^2+^, and Mn^4+^ size-mismatch could further induce local lattice distortions that modify Mn–O–Mn bond angles, affect Jahn–Teller activity thus driving microstructural changes at varying substitution levels. Nano compositions of La(Mn_1−*x*_V_*x*_)O_3_ (*x* = 0, 0.01, 0.03, 0.05) were synthesized by the sol–gel method and thoroughly characterized *via* various techniques. By thoroughly examining the structural, electronic, optical, electrical, and magnetic features and systematically associating the level of substitution with the functional features, this research sets the structure–property relations for vanadium-substituted LaMnO_3_ and determines the best compositions of the material for superior multifunctional capabilities.

## 2. Experimental

### 2.1. Materials and experimental methods

Lanthanum(iii) carbonate hydrate (La_2_(CO_3_)_3_·*x*H_2_O (99.9%), MW 457.85 g mol^−1^) was obtained from Sigma-Aldrich chemical Co., USA. Manganese(ii) nitrate 4-hydrate (Mn(NO_3_)_2_·4H_2_O (99%), MW 251.01 g mol^−1^) from BDH Laboratory Supplies, England. Ammonium meta vanadate (NH_4_VO_3_ (98%), MW 116.98 g mol^−1^) and ethylene glycol (ethane diol LR, C_2_H_6_O_2_ (99%), MW 62.07) were obtained from Research-Lab Fine Chem Industries, India. Citric acid anhydrous (C_6_H_8_O_7_, (99.5%), MW 192.13) from Fisher Scientific, United States. Nitric acid (HNO_3_, 65%) was acquired from Riedel-de Haën.

### 2.2. Preparation of pure and V-substituted LaMnO_3_ nanoparticles

Synthesis of pure LaMnO_3_ perovskite and vanadium-substituted LaMnO_3_ samples were accomplished *via* a modified sol–gel approach. In this process, an α-hydroxycarboxylic acid was used to chelate the metal ion precursors to ensure homogeneous cation distribution at the molecular level. Citric acid was utilized as a chelating agent, while ethylene glycol was used a polymerization promoter for gel formation. Synthesis of LaMnO_3_ (LM) was achieved thorough dissolving stoichiometric amounts of La_2_(CO_3_)_3_·*x*H_2_O and Mn(NO_3_)_2_·4H_2_O separately. Specifically, a mixture of 10 mL nitric acid and 10 mL distilled water was used to dissolve 4.7 g of La_2_(CO_3_)_3_·*x*H_2_O, while 5.2 g of Mn(NO_3_)_2_·4H_2_O was solubilized in 20 mL distilled water. These solutions were subsequently added to a citric acid solution (4.6 g in 100 mL distilled water), then 5 mL of ethylene glycol under stirring to obtain a clear and homogeneous citrate-metal ions chelated solution. The mixture was heated at 85 °C for 12 h with constant stirring to induce dehydration and polycondensation which results in a transparent gel formation. The gel underwent auto-combustion to yield a fine powder. The powder was eventually calcined at 900 °C for 6 h to obtain phase-pure LaMnO_3_. The same procedures were followed to synthesis of vanadium-substituted LaMnO_3_ samples with minor modifications. Predetermined atomic percentages relative to Mn (1, 3, and 5 wt%) of NH_4_VO_3_ aqueous solutions, were introduced into the citric acid solution. The samples were coded LMV1, LMV3, and LMV5, respectively. This approach ensured uniform vanadium-incorporation of into the LaMnO_3_ lattice while the perovskite structure is preserved.

### 2.3. Characterization of materials

XRD patterns were obtained using a PANalytical X'Pert PRO diffractometer with Cu K_α_ radiation (*λ* = 1.5406) operated at (40 kV and 40 mA), scanning over a 2*θ* of (10–80°) with a step size of (0.02°) to investigate the crystalline phases of the samples. The Rietveld refinement of the XRD patterns was carried out using MAUD software. The background was modeled with a polynomial function and refined the instrumental peak-shape function (Pseudo-Voigt/Caglioti parameters) just once on the unsubstituted LMO pattern. This refinement was then kept fixed across the substituted series to maintain a consistent basis for comparing lattice parameters, microstrain, and phase fractions. To ensure the reliability of the refined cell parameters throughout the series, we used two independent approaches to correct the diffractometer zero-point: a global 2*θ*-offset and a sample-displacement correction. The samples' morphology was examined using a scanning electron microscope with energy dispersive spectroscopy and mapping analysis (SEM/EDS, model Quanta 250 FEG), operated at an accelerating voltage of 30 kV.

FTIR transmittance spectra were obtained using Vertex 80 FTIR spectrometer from Bruker Optik GmbH, Germany, using KBr pellet method in the spectral range of 4000–400 cm^−1^ with the resolution of 4 cm^−1^. The optical properties of the samples were measured utilizing UV-vis diffuse reflectance spectroscopy on a JASCO V-570 spectrophotometer equipped with an integrating sphere over the wavelength range of 200–1000 nm. The BG energy was defined from the DRS data using the Kubelka–Munk function and Tauc plot analysis. Room temperature hysteresis loops were got through a vibrating sample magnetometer (VSM, Model LDJ 9600-1, USA) to estimate the magnetization against magnetic field. For dielectric measurements, the synthesized powders were uniaxially pressed into cylindrical pellets (9.5 mm) diameter, (1.5 mm) thickness under a pressure of (20 MPa). The dielectric properties of the prepared samples were investigated using HIOKI Japan 3532-50 LCR HITESTER over a frequency range from 42 Hz to 5 MHz at room temperature.

## 3. Results and discussion

### 3.1. XRD

Fig. 1 shows XRD patterns of pure and V-substituted LaMnO_3_ samples. The distinct Bragg reflections of the pure LaMnO_3_ sample were linked to the trigonal crystal structure (COD no. 1521791, space group *R*3̄*c* (167)). This trigonal *R*3̄*c* phase is stable over the dilute substitution range studied here (1–5% V). This is contrasted to the higher substitution regime (*x* = 0.1–0.9) reported by Teplykh *et al.*^21^ where LaMn_1−*x*_V_*x*_O_3_ substitutes an orthorhombic *Pnma* structure over the whole compositional range studied. This is consistent with the two substitution regimes accessing different structural states of the V-substituted system. Careful examination of the raw patterns for the LMO and LMV1 samples showed no obvious impurity peaks. Weak additional reflections at 2*θ* = 20–30° were observed for the LMV3 and LMV5 samples which are attributed to minor secondary phases (V_2_O_5_ (COD-2102582) and La_2_O_3_ (COD-1531452), discussed below). In addition, all compositions of LaMnO_3_ demonstrate commendable XRD peak intensities, which reflect good crystallinity. V-substituted sample show about the same degree of crystallinity even after the monosubstitution of V at 1% and 3% weight percent. In contrast, a 5% V-substitution sample showed the most significant decrease in crystallinity which was attributed to smaller and less-defined crystalline domains.

**Fig. 1:**
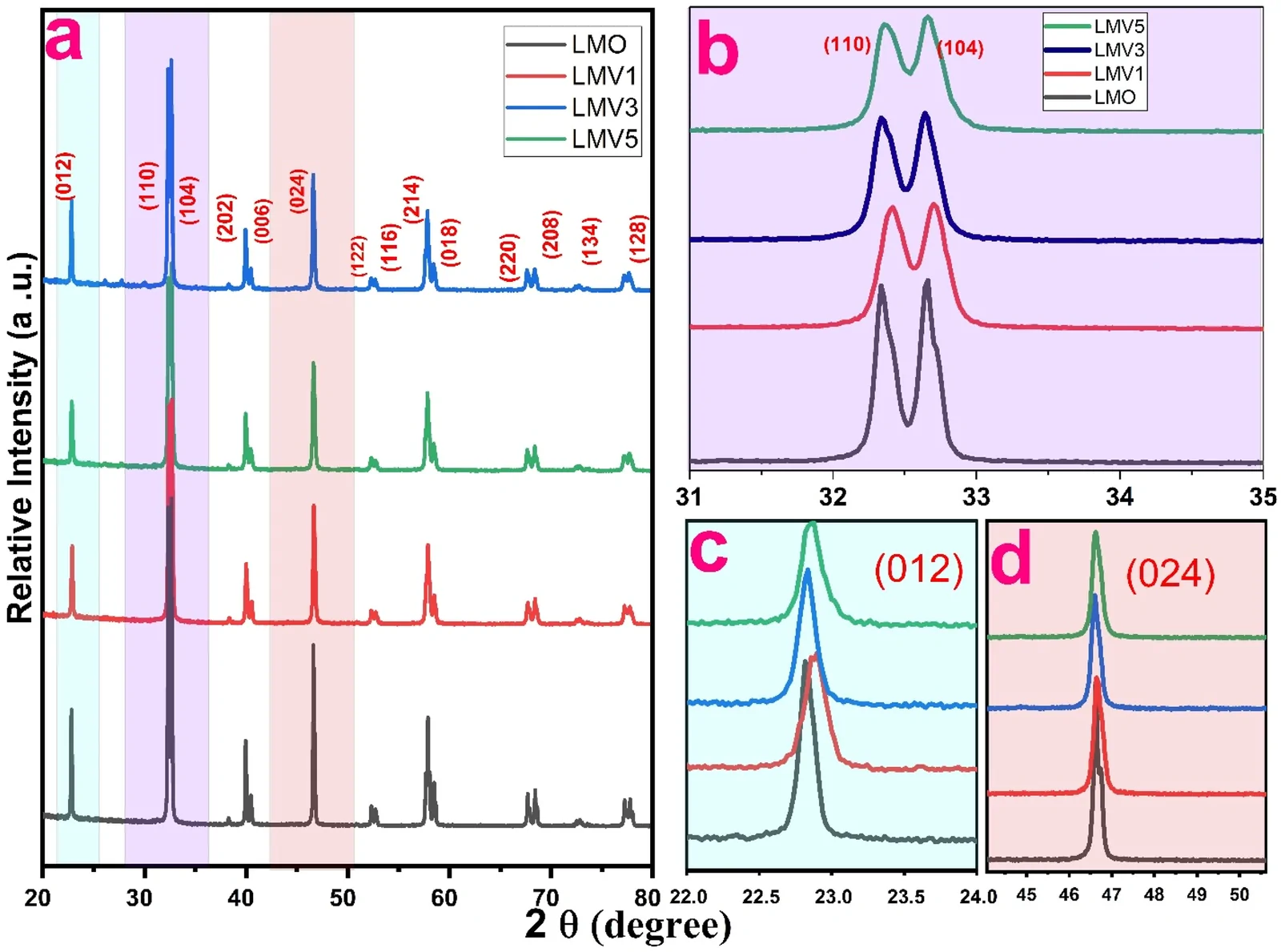
(a) XRD patterns of pristine and V-substituted LaMnO_3_ samples (trigonal *R*3̄*c* phase, COD 1521791), with weak secondary-phase reflections (V_2_O_5_, La_2_O_3_) visible at 2*θ* = 20–30° for LMV3 and LMV5; shifts and changes in the (b) doublet-split principal peaks (110)/(104), (c) (012), and (d) (024) planes.

The effect of V ions-substitution on the LaMnO_3_ lattice was studied by examining the changes in the LaMnO_3_ doublet-split primary peaks found at about 32.5° of (110) and (104) crystal planes, as well as peaks of the (012) and (024) planes located at about 23° and 47° respectively (Fig. 1b–d). shows the results of the effect of V ions on the (110), (104), (012) and (024) planes of the LaMnO_3_ lattice. Clear changes in the positions of 2-theta could mainly be due to the replacement of Mn^3+^ ions by V^*x*+^ ions within these planes.

The systematic investigation shown in Fig. 2 was performed using the least-square Rietveld refinement analysis. It aims to study confirmation of the purity of the phase obtained along with necessary structural data. The results are further denoted in Table 1 and Fig. 3a–d.

**Fig. 2:**
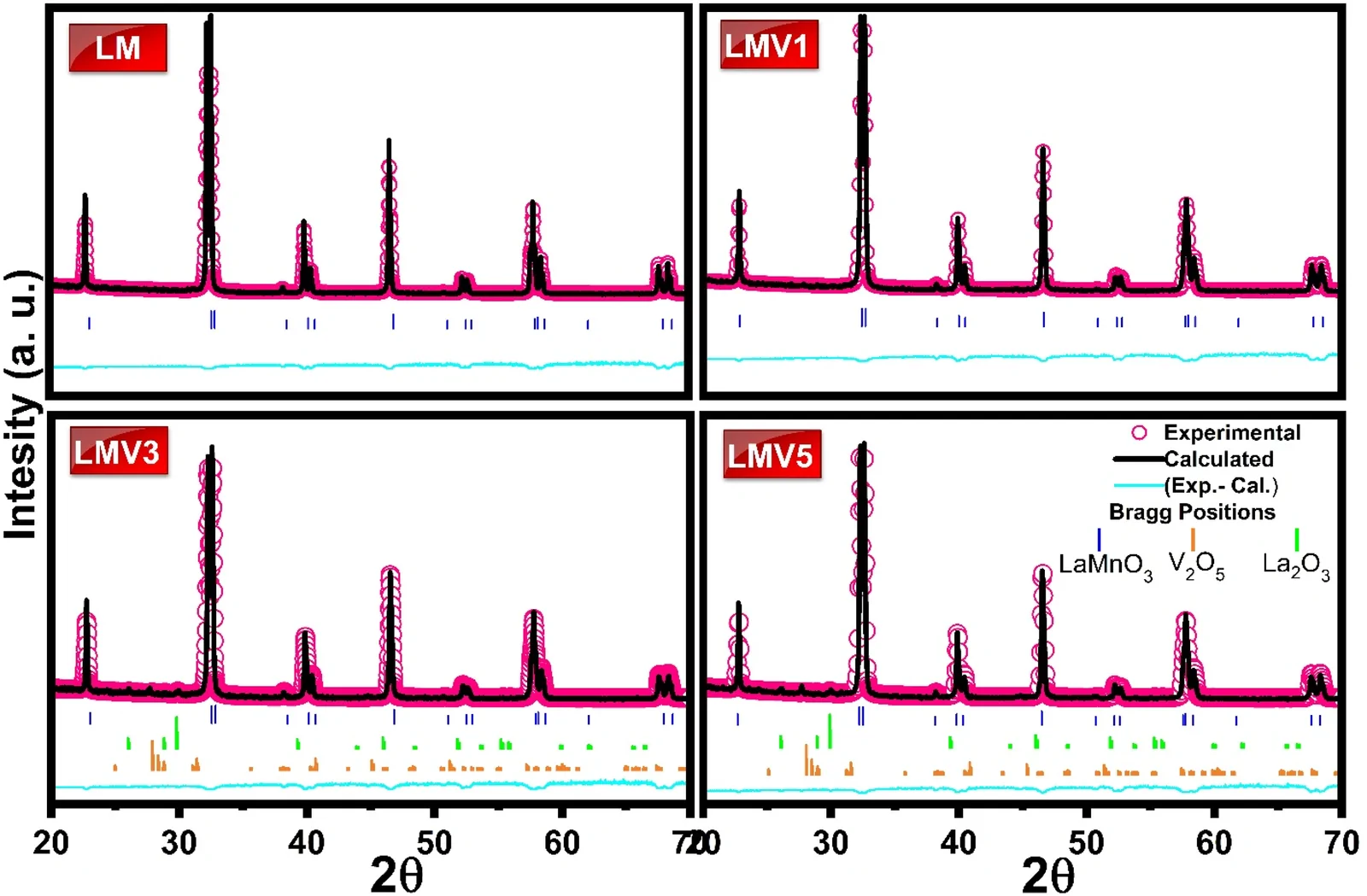
Rietveld refinement patterns of LM, LMV1, LMV3 and LMV5, showing observed, calculated, and difference profiles with Bragg reflection positions marked; refinement statistics are given in Table 1.

**Table 1:** Rietveld refinement parameters, microstrain, phase fractions, and refinement quality values for pristine and V-substituted LaMnO_3_^a^

Parameter	LMO	LMV1	LMV3	LMV5
**Unit cell**				
*a* (Å)	5.5289(9)	5.5334(2)	5.5382(3)	5.5388(2)
*c* (Å)	13.3564(3)	13.3722(6)	13.3829(8)	13.3844(5)
*V* (Å^3^)	353.60	354.61	355.53	355.68
**Microstructure**				
Microstrain (×10^−4^)	0.8(7)	9.5(3)	8.22(2)	8.22(2)
*D*_Scherrer_ (nm)	61.38(2)	45.97(2)	47.63(8)	41.38(5)
**Structural model (space group *R*3̄*c*)**				
La1(*x*, *y*, *z*)	0, 0, 0.25 samples)	0, 0, 0.25	0, 0, 0.25	0, 0, 0.25
La1: Biso (Å^2^)	0.87(5) thereafter)	0.87	0.87	0.87
Mn1/V1: *x*, *y*, *z*	0, 0, 0	0, 0, 0	0, 0, 0	0, 0, 0
Mn1/V1: occupancy (Mn/V)	1.0/0	0.99/0.01	0.97/0.03	0.95/0.05
Mn1/V1: Biso (Å^2^)	0.50	0.50	0.50	0.50
O1: *x*, *y*, *z*	0.4558, 0, 0.25 (fixed, all samples)	0.4558, 0, 0.25	0.4558, 0, 0.25	0.4558, 0, 0.25
**Secondary phases**				
V_2_O_5_ (wt%)	—	—	1.43(62)	1.79(40)
La_2_O_3_ (wt%)	—	—	0.70(11)	0.805(73)
**Refinement quality**				
*R*_wp_ (%)	17.70	14.62	13.71	13.68
*R*_p_ (%)	13.30	10.82	10.01	10.00

a Atomic coordinates and isotropic displacement parameters (*B*_iso_) for La, Mn/V, and O were refined on the LMO pattern and held fixed across the substituted series to isolate lattice-parameter, microstrain, and phase-fraction trends from parameter correlation. Numbers in parentheses are estimated standard deviations on the last digit.

**Fig. 3:**
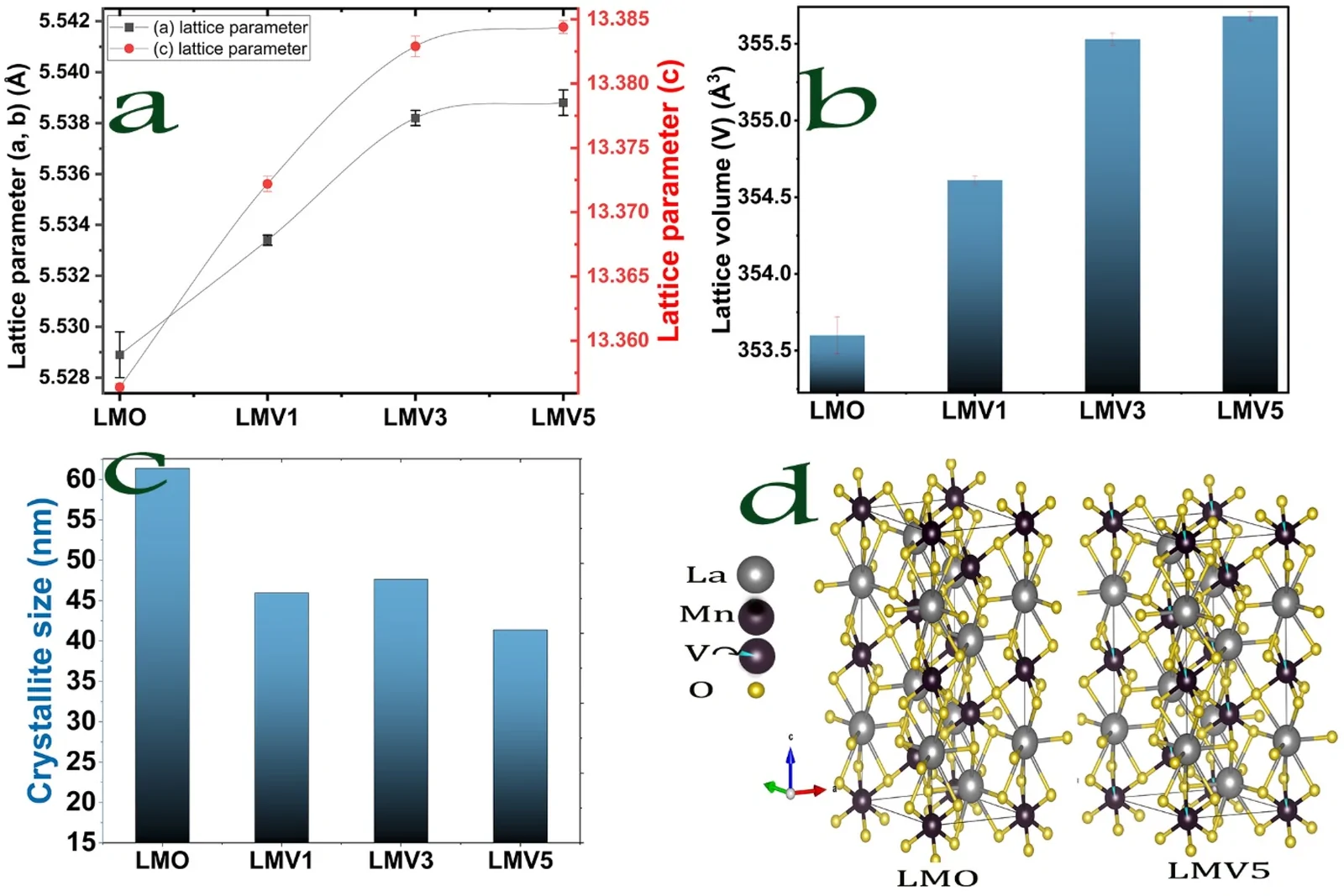
Representation of (a) lattice parameters a and c and (b) unit cell volume V as a function of V-content, with error bars representing e.s.d.s showing the largest increase occurring between LMO and LMV1 and a saturating trend thereafter; (c) apparent crystallite size (Scherrer equation); (d) 3D polyhedral representation of the refined structures for LMO and LMV5.

The refined lattice parameters *a* and *c* increase with V-content as shown in Fig. 3a, b and Table 1, with the largest increase between the pure and 1% samples, and smaller, saturating increases at 3% and 5% substitution. This corresponds to a total unit cell volume expansion of about 0.59% from LMO to LMV5, which is well beyond the estimated uncertainty (Table 1). Interestingly, the expansion rate is significantly reduced after 1% V- substitution, which could be related to the formation of the V_2_O_5_ secondary phase at 3% and 5% (Table 1 and Fig. 3), suggesting that only part of the nominal vanadium content is incorporated into the LaMnO_3_ lattice at higher substitution levels. Fig. 3d displays 3-D packing structures of LaMnO_3_ and vanadium substituted samples which were created from refinement data using VESTA software.

This lattice expansion requires careful interpretation considering the multiple possible oxidation states of both vanadium (V^2+^ to V^5+^) and manganese (Mn^2+^, Mn^3+^, Mn^4+^), each with significantly different ionic radii. Three primary charge compensation mechanisms can explain the observed structural change. First, if vanadium enters as V^2+^ (0.79 Å) replacing Mn^3+^ (0.645 Å), with concomitant Mn^4+^ (0.53 Å) formation for charge balance, the larger V^2+^ would drive lattice expansion.^22^ The second choice is isovalent substitution of V^3+^ (0.64 Å) for Mn^3+^, however such substitution would produce minimal structural change. Third, if vanadium incorporates as V^5+^ (0.54 Å), significantly smaller than Mn^3+^, lattice expansion would only be possible through reduction of Mn^3+^ to Mn^2+^ (0.83 Å). In this scenario, the mean B-site ionic radius increases according to: Δ*r*_avg =_*x*(*r*_V_^5+^ − *r*_Mn_^3+^) + *y*(*r*_Mn_^2+^ − *r*_Mn_^3+^); *x* = fraction of Mn^3+^ replaced by V^5+^, *y* = fraction of Mn^3+^ reduced to Mn^2+^, *r* ionic radium of metal ion. Despite the size reduction from V^5+^ substitution, even modest Mn^2+^ formation would result in net lattice expansion given that Mn^2+^ is 0.185 Å larger than Mn^3+^.

While competing lattice-expansion scenarios cannot be resolved from cell volume alone, the saturating expansion trend (reaching near-maximum by 1% doping) combined with the emergence of a V_2_O_5_ and La_2_O_3_ secondary phase at 3–5% substitution indicates incomplete B-site vanadium incorporation at higher substitutions. Because the V_2_O_5_ (1.43, 1.79 wt%) and La_2_O_3_ (0.70, 0.80 wt%) phase fractions in LMV3 and LMV5, respectively, lie near the practical detection limit of laboratory XRD (1–5 wt%), exact incorporation levels cannot be quantified by XRD alone, and complementary bulk compositional analysis is recommended. This near-detection-limit secondary-phase content is also the likely origin of the anomalous crystallite-size increase at 3% substitution, which interrupts the otherwise monotonic decrease with V content. Furthermore, oxygen non-stoichiometry (vacancies or interstitials) represents an additional degree of freedom that could contribute to charge balance and lattice expansion alongside Mn^3+^/Mn^4+^ redox shifts.

The Debye–Scherrer equation^23–25^ was utilized to calculate the crystallite size through the equation *d* = 0.89*λ*/*β* cos *θ*, which includes 0.89 as a constant and *λ* as the X-ray wavelength and *θ* as the Bragg diffraction angle and *β* as the full width at half maximum of the diffraction peaks. Table 1 displays the results of the calculation, which shows that the addition of V ions caused a significant reduction of average crystallite size, which decreased from its original value of 61.38 nm to 41.38 nm after 5% V substitution was applied. The decrease is not strictly monotonic (Table 1), likely due to some extra uncertainty caused by a secondary phase that's just at the edge of detection at 3% substitution. This trend is corroborated by the independently refined microstrain (Table 1), which also increases with V-substitution, supporting the idea that lattice disorder grows with vanadium substitution. The crystallite size trend is consistent with vanadium ions modifying crystallization behavior, by changing nucleation patterns and growth mechanisms. The V ions function as nucleation sites and growth inhibitors, which explain the reduction of crystallite size that has been documented. The V-presence in the LaMnO_3_ lattice creates local energy variations, which direct the development of tiny crystallites. The nucleation sites reach sufficient abundance at 5% V dopant concentration, which results in the creation of numerous small crystallites, instead of producing a few large ones, according to the observed small crystallite size of 41 nm.^26,27^

### 3.2. SEM

A granular microstructure composed of aggregated nanoparticles was observed in LMO-SEM image (Fig. 4). The individual grains are relatively uniform, with estimated sizes ranging from 0.16 to 0.19 µm. These grains develop tightly packed matrix with minimal pore development. The closely fused arrangement indicates strong interactions between particles during synthesis that resulted in a low-porosity surface morphology. A significant morphological shift is observed in the LMV series upon vanadium ions-incorporation. For LMV1, more open surface with markedly transitioning from the compact granular LMO texture to a highly porous, sponge-like network was observed. This observation was further supported by presence of large voids and hollow regions across the microstructure. This indicates V-substitution at 1% that disrupts the agglomeration of LMO grains and promotes the formation of interconnected macro-pores. These structural openings results in increasing the surface roughness and generally improve mass transfer, adsorption sites, and accessibility for reactant molecules. The LMV3 sample continues this trend of porosity development. Compared to LMV1, the pores became smaller and more interconnected in LMV3, while the walls forming the pore network appear thicker, suggesting suggests a more restructuring controlled effect, where the modifier influences the growth and fusion of grains in a moderated manner. The resulting morphology combines enhanced porosity and improved rigidity of framework, which LMV3 an intermediate structure between the highly porous LMV5 and the compact LM. A further increase in modification, (LMV5), leads to the more developed porous architecture representing the most pronounced structural transformation among the samples. An extensive three-dimensional network of interconnected channels, macropores, and thin skeletal walls resembling a foam-like or coral-like morphology was formed. This high level of pore evolution indicates the strong V-substitution influence at 5% on the nucleation-growth equilibrium which drives the formation of a lightweight, highly accessible structure.

**Fig. 4:**
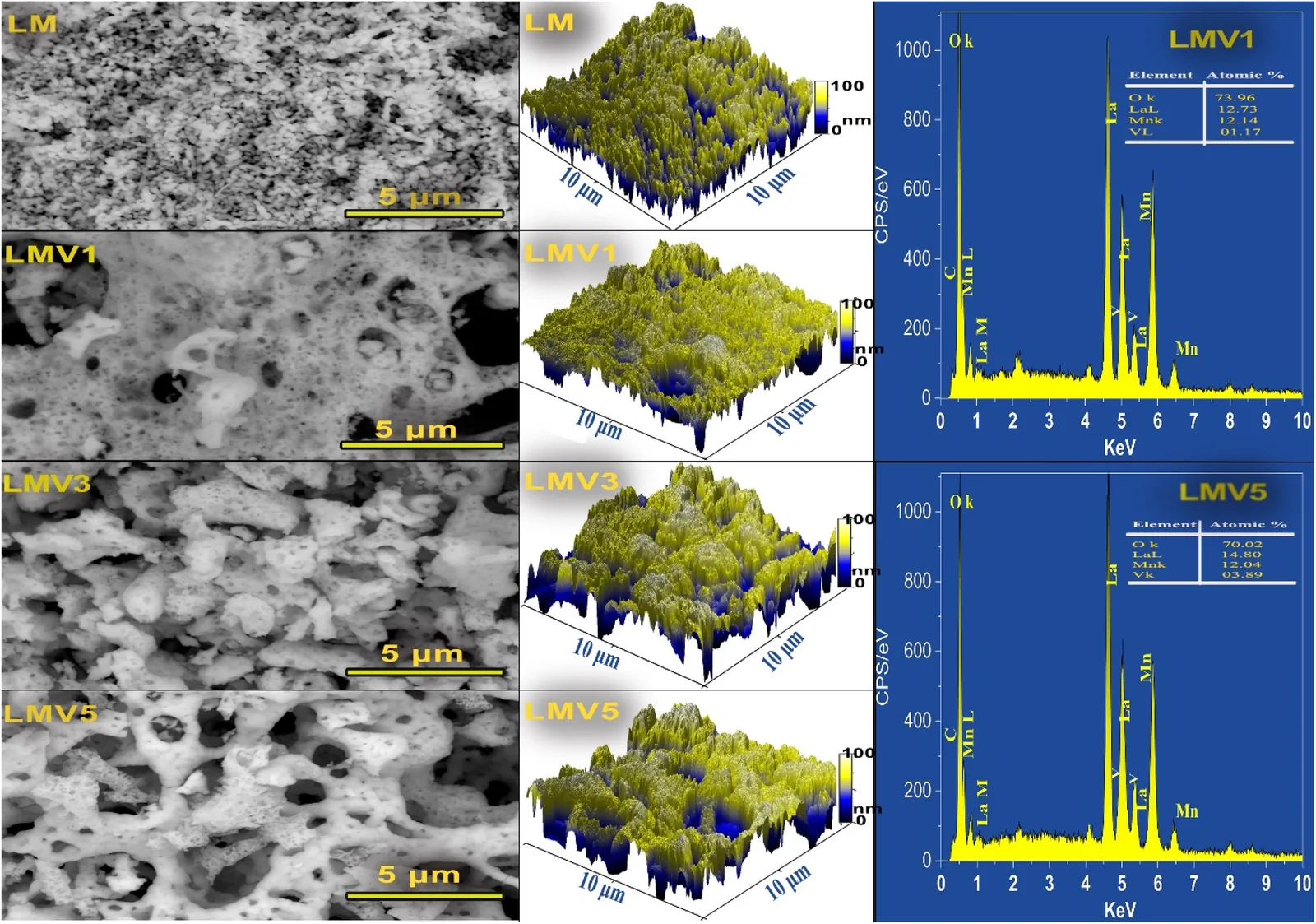
SEM images, 3D surface topography and Energy dispersive spectroscopy (EDX) analysis of LM, LMV1, LMV3 and LMV5, showing the progression from compact grains in LM to a porous, foam-like morphology in LMV5.

The 3D surface topography confirms the SEM observations and shows a clear evolution from the LMO with compact and moderately rough surface to increasingly porous and rugged architectures in vanadium-substituted samples. LMO exhibits closely packed height features with limited depth variation. On the other hand, LMV1 and LMV3 (Fig. 4) show deeper valleys and more pronounced roughness corresponding to the emergence of pore networks. The greatest height contrast and the most irregular topography was detected in LMV5 which is consistent with its highly porous and foam-like morphology. The EDX spectra confirmed the presence of the expected matrix and dopant elements (La, Mn, V, O) without any foreign contamination. The measured increase of the atomic V% from LMV1 to LMV5 is consistent with the expected doping levels, confirming the total vanadium content is consistent with the nominal composition. However, as EDX reports bulk elemental ratios rather than crystallographic phase information, it is not able to distinguish V on the Mn-site from V in the minor V_2_O_5_ secondary phase identified by XRD (Section 3.1).

### 3.3. Optical study

Fig. 5a depicted the diffuse reflectance (DR) spectra of pristine LMO and V-substituted samples (LMV1, LMV3, LMV5). All samples exhibit relatively low R% throughout the entire 200–1000 nm wavelength range which maintains below 50%. This indicates strong light absorption which is characteristic feature of LaMnO_3_ perovskites. The pristine LMO sample shows the lowest *R* across nearly the whole spectrum which reflects its inherently strong absorption related to O^2−^ → Mn charge-transfer (CT) transitions and Mn^3+^ crystal-field excitations. Slightly higher *R*% was shown by V-substituted samples compared to pure LM, particularly in the (200–350 nm) UV region. The modest *R* increasing suggests that V-incorporation alters the electronic structure as well as absorption strength. The highest *R* was exhibited by LMV3, particularly in the UV and near-visible regions which implies that the intermediate V-concentration slightly reduces the efficiency of absorption. In contrast, LMV5 shows the lowest *R* among the V-substituted series which approaches the strong absorption behavior of pristine LM. This suggests that higher substitution percent of V leads to enhancing light-materials interaction, possibly through defect density increasing or/and perturbation of electronic band.

**Fig. 5:**
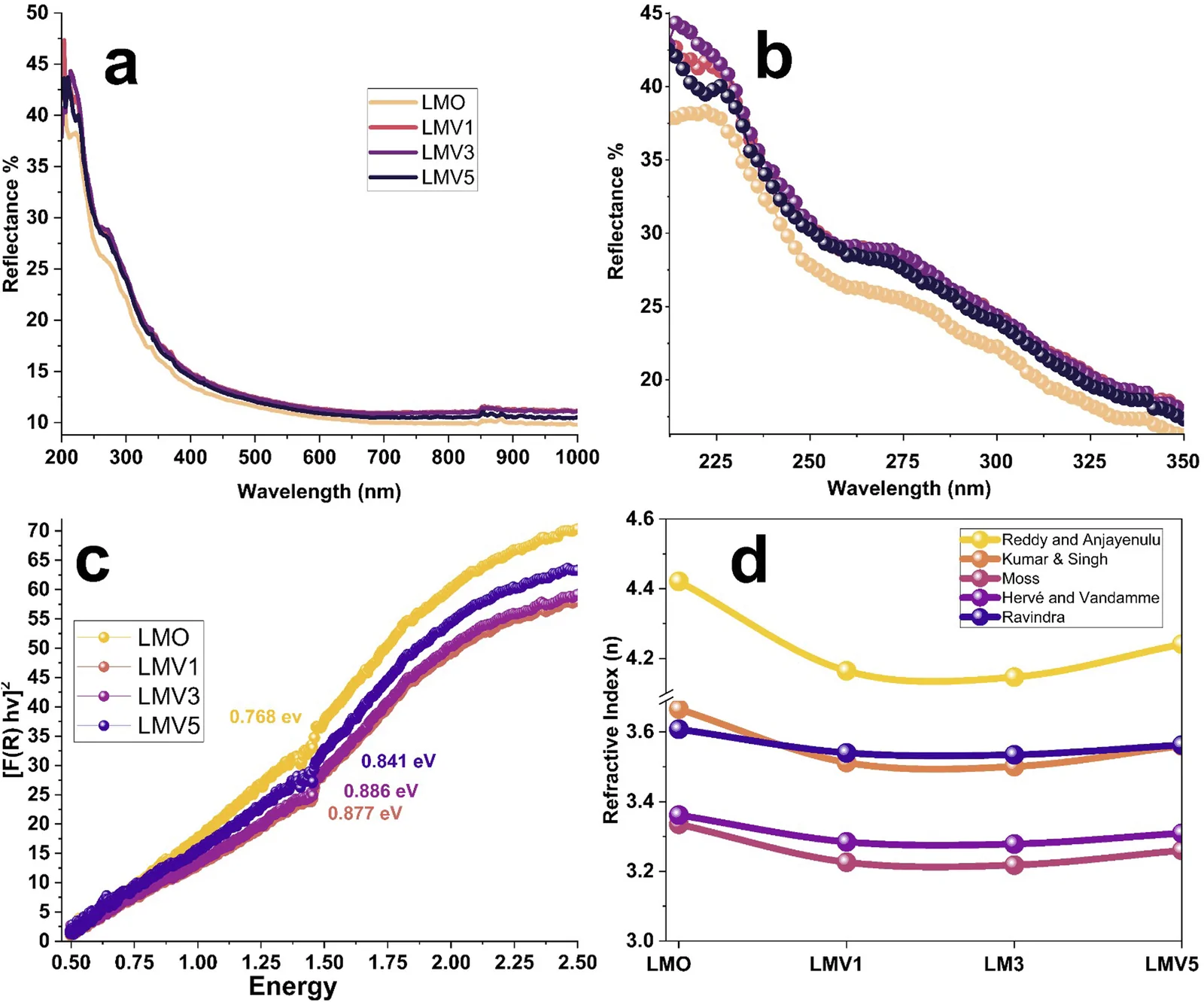
(a) DR spectra, (b) enlarged diffuse reflectance (200–350 nm range), (c) Kubelka–Munk (K–M) plots and BG and (d) representation of refractive index estimated by different models of LM, LMV1, LMV3 and LMV5 samples.

In parallel to these differences observed in the UV, a pronounced drop in *R* (below 15%) was recorded by the all-synthesized samples in the visible-to-NIR region (400–1000 nm). This sharp reduction refers to a highly efficient absorption across the *vis*-spectrum and implies the valuable efficiency of both pristine and V-substituted LMO for harvesting broad range of photon energies. The slight variations among LMV1, LMV3, and LMV5 are consistent with substitution-induced changes in band structure and defect levels.

The spectra show a distinct, relatively sharp incline in the UV-visible region that corresponds to the fundamental absorption edge of LaMnO_3_. This edge becomes both sharper and shifted to higher photon energy (blue shift) for the V-substituted samples up to LMV3, indicating an increase in the effective optical BG. To quantify this, we converted the reflectance (%) to the Kubelka–Munk remission function (Fig. 5c), *F*(*R*) = (1 − *R*)^2^/2*R*, and applied a Tauc analysis by plotting [*F*(*R*)*hν*]^*n*^*vs. hν* and extrapolating the linear portion to intercept the energy axis,^28,29^ yielding band-gap energies of 0.768 eV (LM), 0.877 eV (LMV1), 0.886 eV (LMV3) and 0.841 eV (LMV5). Physically, the initial blue shift (from LMO to LMV3), manifested as increased BGs for LMV1 and LMV3, can be rationalized by two complementary mechanisms. Firstly, V-substitution at the B-site perturbs the Mn–O–Mn network as the V-ions with their higher oxidation tendency modifies the Mn^3+^/Mn^4+^ balance and reduces the bandwidth of the Mn-derived conduction/valence bands. This reduced bandwidth increases the energy separation between occupied and unoccupied states and produces an apparent optical gap widening. Secondly, partial electron depletion of Mn d-states by higher-valence V can shift the Fermi level and reduce available low-energy transitions; if carrier filling of the conduction band increases, a Burstein–Moss-type effect can also produce an apparent blue shift of the absorption edge.^2,30,31^ Both structural (bandwidth) and carrier-population effects therefore act to increase the measured gap at low-to-moderate V concentrations. At the highest substitution (LMV5) the BG decreases slightly (from 0.886 to 0.841 eV) but still higher than the pristine sample LM. This partial reversal is consistent with the formation of additional defect and impurity states inside the gap when V concentration becomes large. Increased disorder, oxygen-vacancy formation, or V-related localized states produce band-tailing and mid-gap levels that allow sub-bandgap optical transitions, which reduce the apparent optical gap. This interpretation is supported by the reflectance behaviour: LMV5 shows the strongest overall absorption (lowest *R*%) among the substituted sample, a signature that inhomogeneous broadening and defect-mediated absorption become important at higher dopant levels.

The V_B_ and C_B_ edge of La(Mn_1−*x*_V_*x*_)O_3_ were assessed utilizing Mulliken relations^32–34^ (Fig. 6), *E*_VB_ = *X* − *E*_e_ + 0.5 *E*_g_ and *E*_CB_ = *E*_VB_ − *E*_g_. For pristine LaMnO_3_, the calculated C_B_ and V_B_ were 1.10 eV and 1.87 eV, respectively. Upon V-substitution, subtle shifts were observed. At low substitution (*x* = 0.01), a modest widening of the band separation was observed as the C_B_ edge decreased slightly to 1.05 eV while the V_B_ rose to 1.93 eV. For *x* = 0.03, a downward shift of the CB (1.03 eV) was accompanied by a V_B_ at 1.92 eV which reflects continued electronic structure modification *via* V-substitution. At higher loading (*x* = 0.05), a C_B_ increasing (1.06 eV) and a V_B_ decreasing (1.90 eV) was detected which suggests a partial rebalancing of band-edge alignment at elevated V-content. These trends demonstrate that substitution of Mn ions by V^5+^ perturbs the electronic network of Mn–O–Mn and induces measurable band-edge energies adjustments, which is consistent with the observed variations in optical absorption and BG widening.

**Fig. 6:**
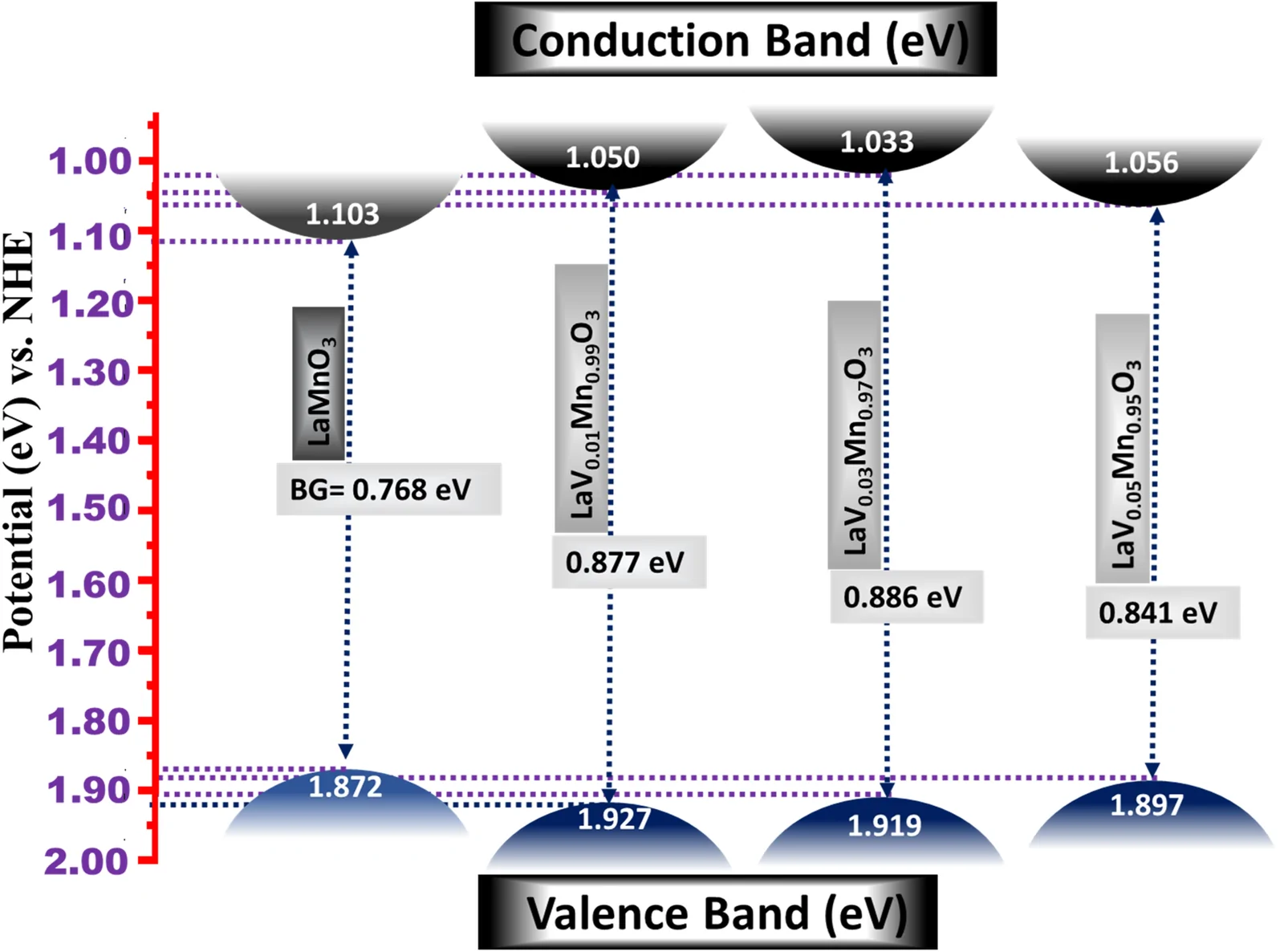
The valence band and conduction band edge positions of LM, LMV1, LMV3 and LMV5 calculated *via* Mulliken electronegativity relations, showing band-edge shifts with V-content consistent with the optical band-gap trend.

The refractive index (*n*) (Fig. 5d) of the LMO and V-substituted samples was estimated using the Reddy–Anjaneyulu, Kumar–Singh, Moss, Hervé–Vandamme, and Ravindra empirical models (Table 2).^35,36^ The pristine LMO sample exhibited the highest refractive index (3.33–4.42) which is consistent with their formulations BG value (*E*_g_ = 0.768 eV). Upon V-substitution, the BG increases to 0.877 eV (LMV1) and 0.886 eV (LMV3) which leads to a clear reduction in the refractive index to approximately 3.23–4.16 for LMV1 and 3.22–4.15 for LMV3, reflecting the electronic polarizability decline and reduced density of low-energy electronic transitions. Interestingly, the refractive index shows a partial recovery at the higher substitution level (LMV5), with values of 3.26–4.24 still below the pristine LaMnO_3_ range (3.33–4.42) across all five empirical models. This shift suggests that vanadium incorporation at 5% modifies the electronic structure *via* changes in Mn–O–Mn bond angle and increased lattice polarization.^37,38^

**Table 2:** Refractive index of LaMnO_3_ and vanadium substituted-LaMnO_3_ and estimated by different models

Sample	Band gap	Reddy & Anjaneyulu	Kumar & Singh	Moss	Hervé–Vandamme	Ravindra
LMO	0.768 ± 0.005	4.42 ± 0.0127	3.67 ± 0.0071	3.34 ± 0.0050	3.36 ± 0.0034	3.61 ± 0.0029
LMV1	0.877 ± 0.004	4.16 ± 0.0080	3.51 ± 0.0051	3.23 ± 0.0036	3.28 ± 0.0027	3.54 ± 0.0024
LMV3	0.886 ± 0.004	4.15 ± 0.0072	3.50 ± 0.0046	3.22 ± 0.0033	3.28 ± 0.0025	3.53 ± 0.0023
LMV5	0.841 ± 0.004	4.24 ± 0.0093	3.56 ± 0.0057	3.26 ± 0.0040	3.31 ± 0.0029	3.56 ± 0.0026

### 3.4. Infrared spectra

Fig. 7 shows the FTIR spectra of pristine and V-substituted LaMnO_3_ samples. In the pristine LaMnO_3_, the main absorption centered at 622 cm^−1^ is ascribed to *ν*_s_ of Mn–O bonds of MnO_6_ octahedra, while the band near 433.7 cm^−1^ corresponds to the O–Mn–O *δ*-mode.^39,40^ These two bands represent the fundamental lattice vibrations of the perovskite framework and confirm MnO_6_ octahedral network formation. Upon V-substitution, shifts in the Mn–O stretching band toward lower wavenumbers (609 cm^−1^) are observed in LMV1, LMV3, and LMV5 which refer to modification of the BO_6_ octahedral environment. This red shift reflects that V^5+^ incorporation induces bond strength and bond length variations in the Mn-sites perturbing the Mn–O–Mn exchange network. Such perturbations reduce the effective stiffness of Mn–O bonds and introduce local octahedral distortion. This is consistent with structural and electronic findings inferred by XRD analysis. In contrast, the O–Mn–O bending mode remains nearly unchanged across all compositions which confirms preservation of long-range perovskite framework despite vanadium-B-site substitution. The coexistence of shifts in the stretching mode and stable bending mode demonstrates that vanadium-substitution induces controlled local distortion rather than degradation of structural. Additional absorption features are observed in all samples. The broad band observed around 3428.5 cm^−1^ is assigned to O–H stretching vibrations related to surface-adsorbed H_2_O or OH groups. While the band around 1633.8 cm^−1^ corresponds to H–O–H bending and residual C–O-related vibrations.^41–43^ Weak bands at 2921.3 and 2850.9 cm^−1^ are attributed to CH_2_ stretching modes which indicates presence of trace organic remnants.^44^

**Fig. 7:**
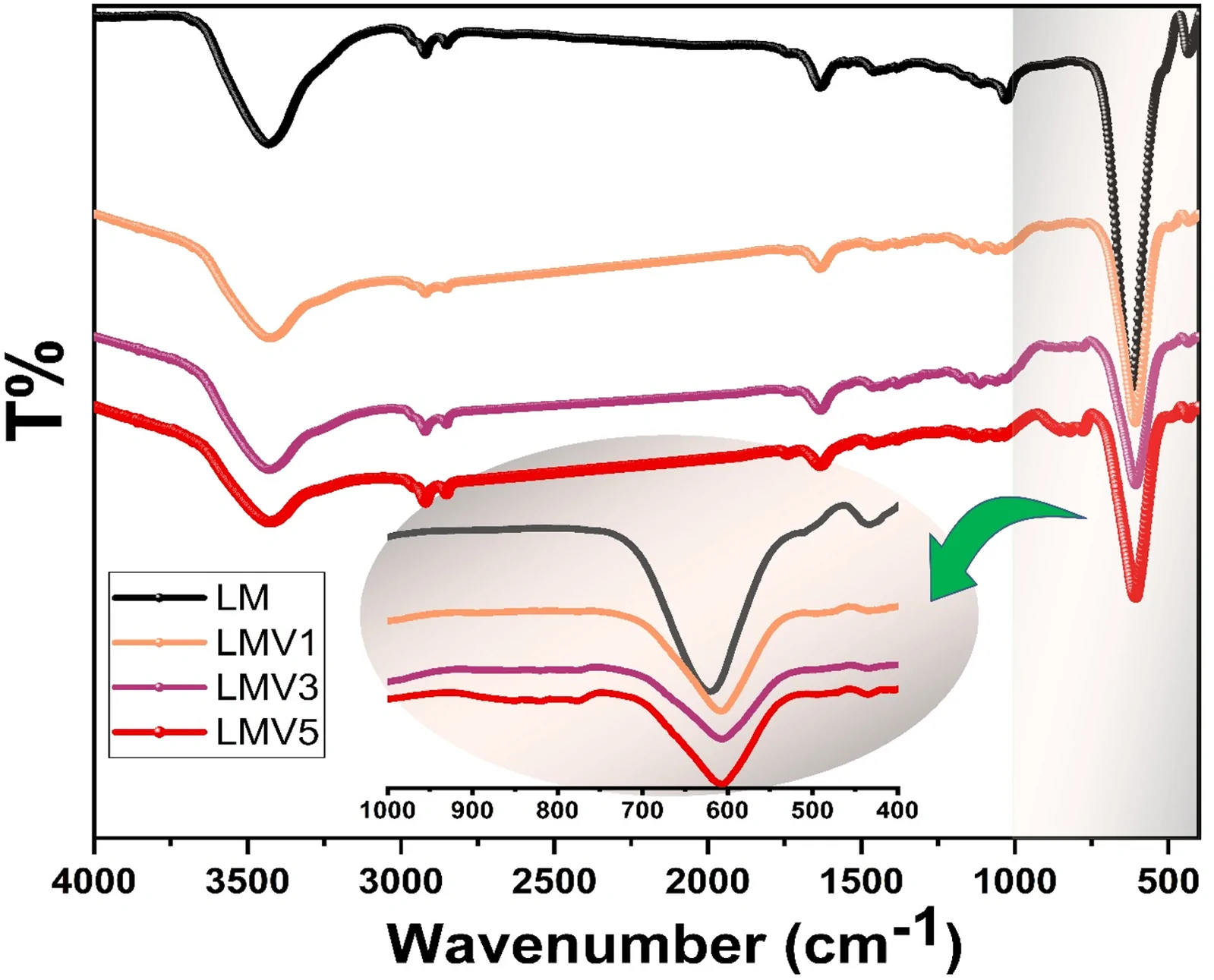
Infrared spectra of LM, LMV1, LMV3 and LMV5, (inset) magnified 400–1000 cm^−1^ region showing the Mn–O stretching band shift from 622 to 609 cm^−1^ with V-substitution, while the O–Mn–O bending band remains nearly unchanged.

### 3.5. Dielectric properties

The dielectric constant (*ε*′) of pristine and vanadium-substituted LaMnO_3_ samples (Fig. 8a) exhibit apparent variations depending on both frequency and substitution concentration. At a given frequency, noticeable variations in (*ε*′) was observed with V-substitution percentage that follows the trend: LMO < LMV3 < LMV5 < LMV1. For instance, at 100 Hz, the dielectric constant increases from 367.07 in pristine LaMnO_3_ to 4793.91, 2241.86, and 3239.65 upon V-substitution at 1%, 3%, and 5%, respectively. This represents an enhancement of approximately 13-fold for the 1% substituted sample compared to the pristine material. The dramatic increase in (*ε*′) upon substitution of vanadium can be attributed to several consistent mechanisms. First, V^5+^ ions-introduction of into the Mn^3+^ lattice sites creates charge imbalance which could be compensated through establishment of mixed-valence manganese states (Mn^2+^/Mn^3+^/Mn^4+^). This mixed-valence configuration facilitates hopping of electrons between different Mn oxidation states (particularly Mn^3+^ → Mn^4+^). This is consequently enhances space charge polarization and interfacial polarization effects.^29,45^ The increasing in charge carriers and defect states (oxygen vacancies or Mn^2+^ centers) lead to formation of more polarization centers contributing significantly to the dielectric response, especially at lower frequencies. Secondly, XRD analysis reported lattice expansion observed upon V-substitution; this may facilitate ionic displacement and ionic polarization enhancement. Thirdly, the structural distortion induced by V^5+^ (0.54 Å) and Mn^3+^ (0.645 Å) size mismatch, combined with the possible presence of larger size Mn^2+^ (0.83 Å) lead to creation of local structural disorders. These disorders can trap charges at grain boundaries and domain interfaces and boost the interfacial polarization.^36,46^ Interestingly, *ε*′ does not vary monotonically with vanadium content, and notably follows a trend inverse to the AC conductivity discussed below (*σ*: LMV3 > LMV5 > LMV1 ≫ LMO). This is mechanistically consistent: *ε*′ and *σ* arise from the same substitution-induced charge carriers but reflect competing fates, interfacial trapping (favoring polarization) *versus* delocalized hopping (favoring conduction). It is noted that LMO, which lacks V-induced mixed-valence Mn states and associated defect/carrier population, shows both the lowest *ε*′ and *σ* among all samples. It is not involved in the inverse *ε*′/*σ* relationship, which instead reflects how the same V-doping-generated carrier population partitions between interfacial trapping and delocalized hopping across LMV1, LMV3, and LMV5. Furthermore, the V_2_O_5_ and La_2_O_3_ secondary phases detected in LMV3 and LMV5 (Table 1) might also influence the dielectric response: preferentially located at the grain boundaries, with a different intrinsic permittivity from the LaMnO_3_ matrix, these phases could modulate the extent of the interfacial polarization and/or the tortuosity of the current path, in addition to the carrier trapping-delocalization mechanism. At 1% substitution, defect/mixed-valence Mn density is sufficient for space-charge and Maxwell–Wagner–Sillars trapping but insufficient for a connected hopping network; consistent with this, no V_2_O_5_/La_2_O_3_ secondary phase is detected at this composition (Table 1), and vanadium remains incorporated as isolated point defects, maximizing carrier localization and *ε*′. At 3% substitution, higher defect density crosses into a percolating hopping regime, favoring carrier delocalization: *σ* maximizes while *ε*′ drops. With 5% substitution, porosity increases (SEM) and leads to more insulating pore-grain interfaces that partly restore interfacial trapping effects (*ε*′ recovered) with a reduced effective conduction cross-section and increased tortuosity, thereby resulting in lower *σ* than LMV3. The competing effects of carrier trapping and delocalization, which are controlled by the evolving microstructure/defect structure along the series, can self-consistently explain the non-monotonic, mutually inverse behaviors of *ε*′ and *σ*. The (*ε*′) frequency dependence shows the characteristic dispersion behavior typical of perovskite manganites, with (*ε*′) decreasing monotonically as frequency increases. For example, in the LMV1 sample, (*ε*′) decreases from 4793.91 at 100 Hz to 59.83 at 1 MHz. This strong frequency dispersion is a hallmark of dielectric relaxation in heterogeneous materials and can be well-described by both Koops' phenomenological model^47,48^ and the Maxwell–Wagner–Sillars (MWS) models.^49^ These models attribute the behavior to slower polarization processes, such as space charge and MWS interfacial polarization. This process can significantly contribute at low frequencies but as frequency increases, the rapidly alternating field hinders their contribution and leads to the observed decrease in (*ε*′).

**Fig. 8:**
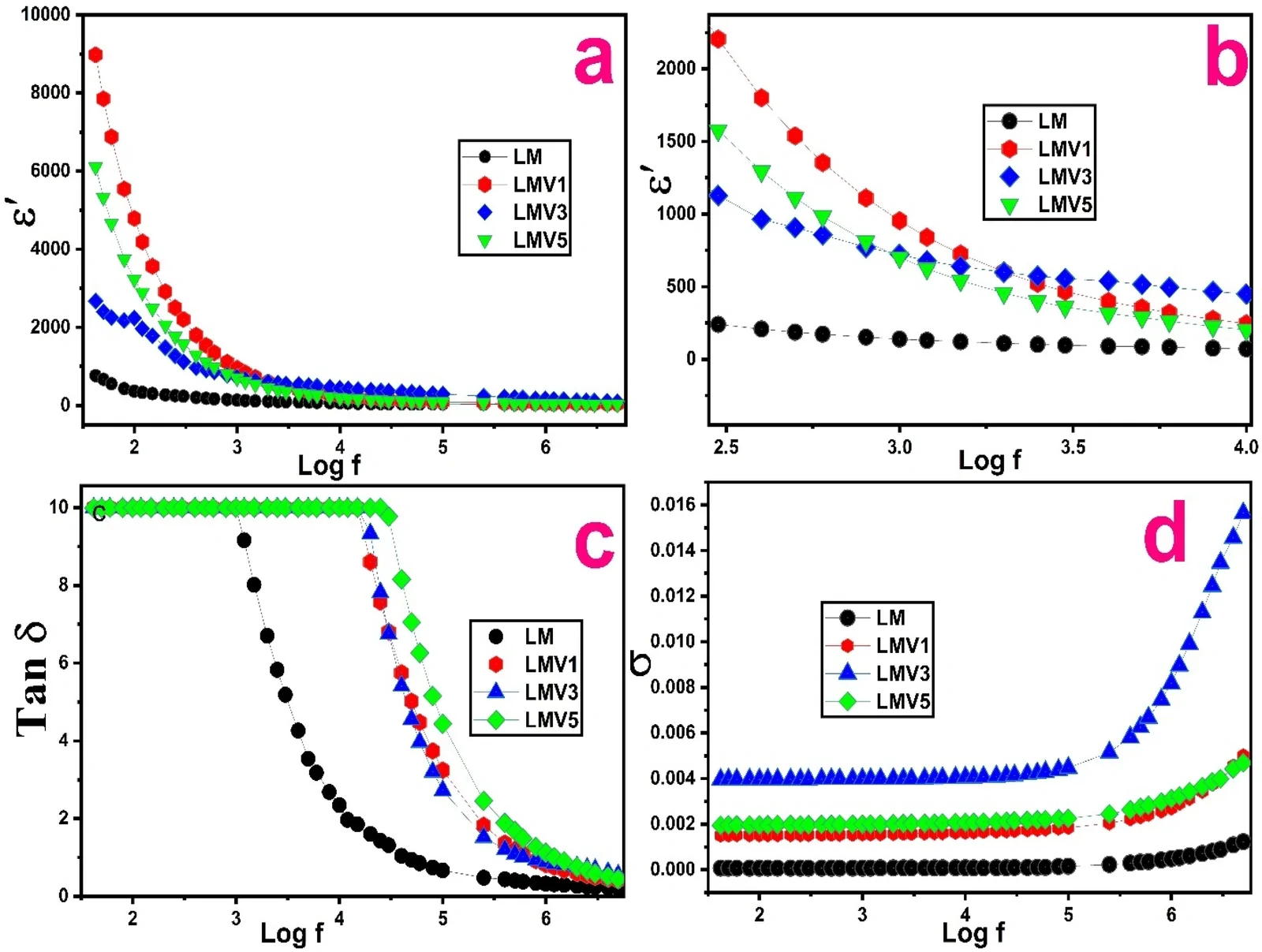
Variation of (a) dielectric constant (*ε*′), (b) zoom region enlarged dielectric constant, (c) tan *δ* and (d) *σ*_ac_ (S cm^−1^) as a function of frequency for LM, LMV1, LMV3 and LMV5 samples.

The tan *δ* curves (Fig. 8c) of LMO and V-substituted LMV samples reveal characteristic decreases in dielectric loss with frequency increasing. At low frequencies, significant energy dissipation was observed in all samples represented by the very high tan *δ* values (about 10). This behavior is typical for dielectric systems related to the domination of interfacial and space-charge polarization at low frequencies which causes substantial lag in dipoles-follow to the external field.^50,51^ As frequency increases (log *f* ≈ 3.5–5.2), tan *δ* drops sharply in all samples. This is reflecting the sluggish charge carriers and dipoles inability to respond to the rapidly oscillating field. However, the onset and rate of this tan *δ*-decrease vary between samples. The earliest and fastest tan *δ* decline was shown by LMO, while a delayed tan *δ*-decrease was observed in LMV1 and LMV3, which suggest higher defect concentration or enhanced hopping polarization due to incorporation of V ions. LMV5 demonstrates the slowest tan *δ*-decline, and retains high tan *δ* values up to higher *f*. This indicates to creation of increased charge carrier density and additional localized states at higher V-content. At high frequencies (log *f* > 6), all samples converge toward low tan *δ* (∼0.1–0.3), where electronic polarization is the only contributor, and losses become minimal. These findings confirms that vanadium-substitution enhances charge carriers localization and polarization defects centers contributing to higher tan *δ* loss at low-frequencies.^51,52^ The convergence of tan *δ* at high frequencies indicates that both pure and V-substituted LaMnO_3_ compositions reach an intrinsic polarization regime dominated by lattice response.

(Fig. 8d) shows that the conductivity (*σ*_ac_) of V-substituted LaMnO_3_ samples displays dramatic enhancement compared to pristine material. At 100 Hz, (*σ*_ac_) increases from 7.42 × 10^−5^ S cm^−1^ in pristine LaMnO_3_ to 0.00153, 0.00396, and 0.00198 S cm^−1^ for 1%, 3%, and 5% V-substitution respectively which represents enhancements of 20-fold, 53-fold, and 27-fold. The (*σ*_ac_) follows a non-monotonic trend: LM3 (3%) > LM5 (5%) > LMV1 (1%) >> LM. This trend is contrast with the (*ε*′) behavior where 1% substitution showed maximum enhancement. This indicates that the mechanisms governing charge transport differ from those controlling polarization. The (*σ*_ac_) enhancement originates from mixed-valence manganese states (Mn^2+^/Mn^3+^/Mn^4+^). This enable polarons hopping through the double-exchange mechanism: Mn^3+^–O^2−^–Mn^4+^ → Mn^4+^–O^2−^–Mn^3+^.^53,54^ Thus, vanadium substitution increases the density of charge carriers by Mn^3+^/Mn^4+^ ratio and modulating and creating of additional hopping pathways. The LMV3 sample exhibits the maximizing (*σ*_ac_) indicating optimal balance between carrier density and mobility. Both pure and V-substituted samples display positive frequency dispersion, with increasing of conductivity (*σ*_ac_) with frequency. For example, LMV3 recorded an increase in (*σ*_ac_) from 0.0040 S cm^−1^ at 100 Hz to 0.016 S cm^−1^ at ∼1 MHz which is nearly 4-fold enhancement. The frequency dependence is consistent with the correlated barrier hopping (CBH) model or small polaron hopping mechanism.^55,56^ The non-monotonic substitution trend is suggesting competing effects. At 3%, optimal carrier density is achieved with sufficient hopping pathways, while at higher substitution levels (5%) excessive defect scattering begins to impede mobility which slightly reduces conductivity relative to the 3% sample. Critically, at 5% V-substitution extensive porosity was revealed by the LMV5 (SEM). This porous morphology drastically reduces the effective cross-sectional area for charge transport. It also introduces air gaps acting as insulating barriers and forcing current to flow through tortuous paths with significantly increased effective length.^57,58^ The complementary behavior of (*ε*′) showing maximum at 1% V-substitution and (*σ*_ac_) with maximum at 3% V-substitution indicates the possibility of application-specific optimization is. LMV1 suits energy storage, needs high *ε*′, lower *σ*, while applications requiring charge transport such as electrochemical devices favor LMV3.

The frequency dependence of the impedance real (*Z*′) and imaginary (*Z*″) of LaMnO_3_ (LM) and V-substituted (LMV1, LMV3 and LMV5) is illustrated in Fig. 9. In all cases, the real impedance (*Z*′) displays a apparent diminution with frequency rising which reflects change from dominated grain-boundary resistance at low frequency to enhanced charge mobility and bulk conduction at higher frequency. *Z*″ exhibits a distinct relaxation peak, corresponding to processes of dipolar and space-charge polarization. Notably, the shift of this peak in position and intensity varies upon changing the V-substitution concentration. This indicates variations in relaxation time and charge-carrier dynamics. From LMO to LMV3, both *Z*′ and *Z*″ decrease significantly, and the *Z*″ peak shifts to higher frequencies. This confirms electrical conductivity improvement and faster relaxation due to more efficient localized charge carriers hopping.

**Fig. 9:**
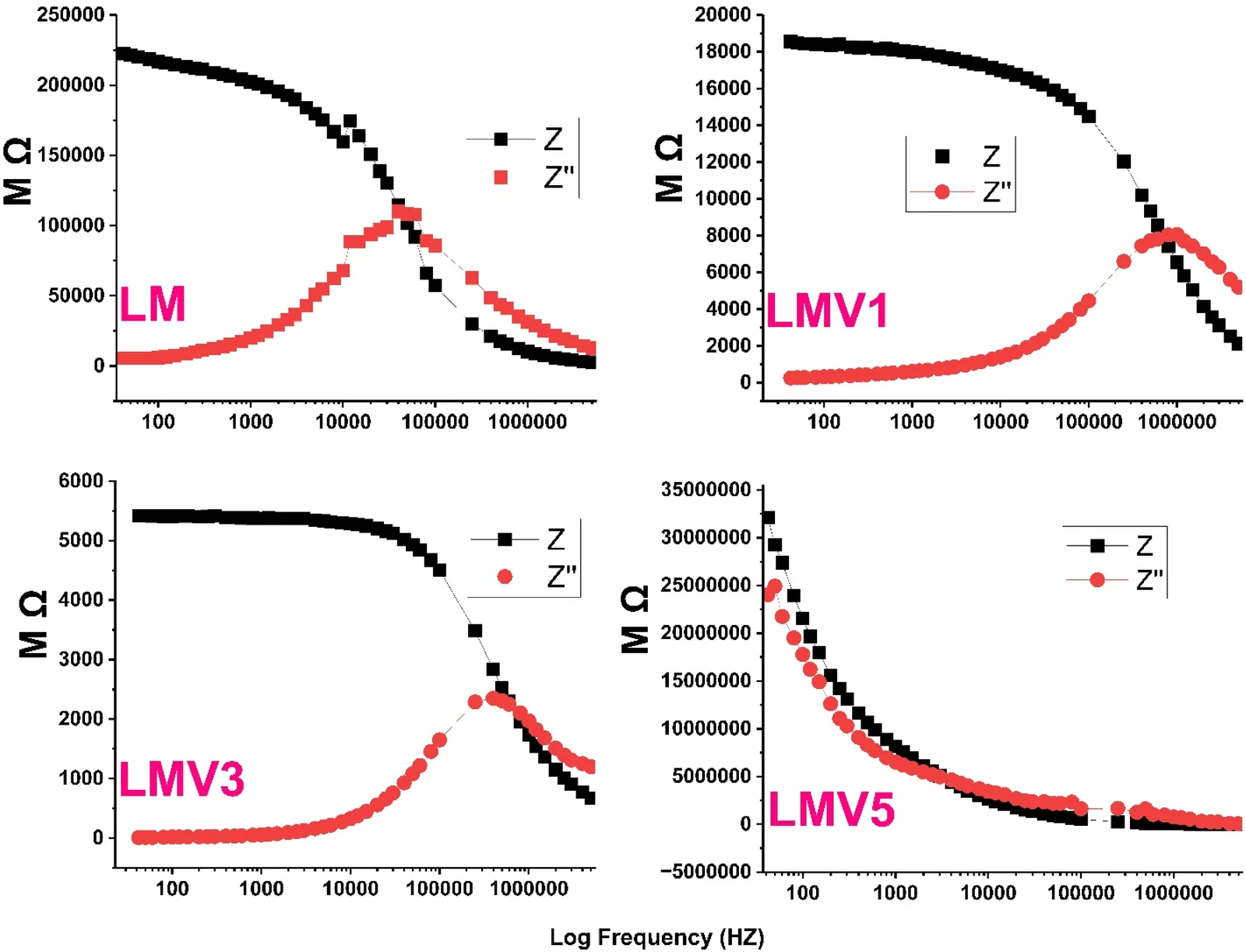
Variation of real (*Z*′) and imaginary (*Z*″) components of the impedance as a function of frequency for LM, LMV1, LMV3 and LMV5 samples showing the *Z*″ relaxation peak shifting to higher frequency with V-substitution up to LMV3, and a broadened, suppressed relaxation response in LMV5.

Large *Z*′ variations are observed in the low frequency range among all samples. LMO begins at 2.2 × 10^5^ Ω, LMV1 at ∼1.8 × 10^4^ Ω, and LMV3 at ∼5.5 × 10^3^ Ω which confirms a progressive *σ*_ac_ enhancement from LMO to LMV3. The imaginary part (*Z*″) displays clear Debye-like relaxation peaks, with LMO peaking near 1 × 10^4^ Hz at 1.1 × 10^5^ Ω. The V-substituted samples revealed upward shifts in relaxation frequency and continuous reduction in *Z*″ magnitude which indicates shorter relaxation times and more efficient dipolar and space-charge polarization; this is consistent with the charge-carrier hopping improved within the lattice.^59,60^ In contrast, distinctly different behavior is shown by LMV5. *Z*′ starts at extremely high value (3.2 × 10^6^ Ω) then decreasing slowly which signifies strong blocking effects and limited charge mobility. Its *Z*″ response is broad and weak, lacks the sharp Debye-like peak. The relaxation maximum appears heavily suppressed and extended across a broad frequency range instead of concentrated near a single relaxation time. This suggests non-Debye distributed relaxation associated with clustered defects or highly resistive grain boundaries which is consistent with the LMV5 micro-porous architecture.^61^

### 3.6. Magnetic properties

Fig. 10 depicts the magnetic hysteresis loops of the pristine LaMnO_3_ and the V-substituted samples. Pristine LaMnO_3_ exhibits characteristic magnetic behavior consistent with the well-documented electronic structure of LaMnO_3_. Characteristic narrow S-shaped loop with relatively weak magnetization (*M*_s_ = 2.91 emu g^−1^), low coercivity (*H*_c_ = 72 G), and modest remanence (*M*_r_ = 0.318 emu g^−1^) are observed (Table 3). LaMnO_3_ is structurally and magnetically polymorphic. Stoichiometric, orthorhombic LaMnO_3_ is an A-type antiferromagnetic insulator with Néel temperature *T*_N_ ≈ 140 K established by neutron diffraction and spin-wave measurements^62,63^ and is therefore expected to be paramagnetic at 300 K. Oxygen non-stoichiometry (LaMnO_3+*δ*_) creates extra Mn^4+^*via* cation vacancies and induces a transition to a rhombohedral phase which orders ferromagnetically instead, with the ordering temperature rising from *T*_N_ = 135 K (*δ* = 0.00) to *T*_C_ = 240 K (*δ* = 0.18), and *T*_C_ ≈ 170–180 K reported for the intermediate range 0.05 ≤ *δ* ≤ 0.11.^10,64^ LaMnO_3_ also adopts a pseudo-cubic phase above the Jahn–Teller transition (∼750 K) while no monoclinic polymorph of the parent compound has been reported;^10,65^ neither is relevant to measurements at 300 K. In a study spanning a comparable *δ* range (0.11–0.26), *T*_C_ values narrows from ∼154 K to ∼142 K as Mn^4+^ content rises before collapsing into spin-glass-like behavior at still higher Mn^4+^ content.^65^ The hysteresis observed here at 300 K thus indicates a ferromagnetic-like contribution in addition to the bulk antiferromagnetic phase and could be attributed to two non-exclusive effects. The first is surface spin canting/uncompensated moments, a size dependent effect reported for antiferromagnetic manganite nanoparticles of similar dimensions to those presented here (Table 1, *D* = 41–61 nm), which results in a hysteretic surface response which remains above the bulk ordering temperature of the particle core.^66^ The second is short-range ferromagnetic clustering: aliovalent substitution of Mn^3+^ for V^5+^ necessitates local charge compensation, which is expected to yield mixed Mn^3+^/Mn^4+^ (and possibly Mn^2+^) valence states, the compositional precondition for double-exchange coupling.^67,68^

**Fig. 10:**
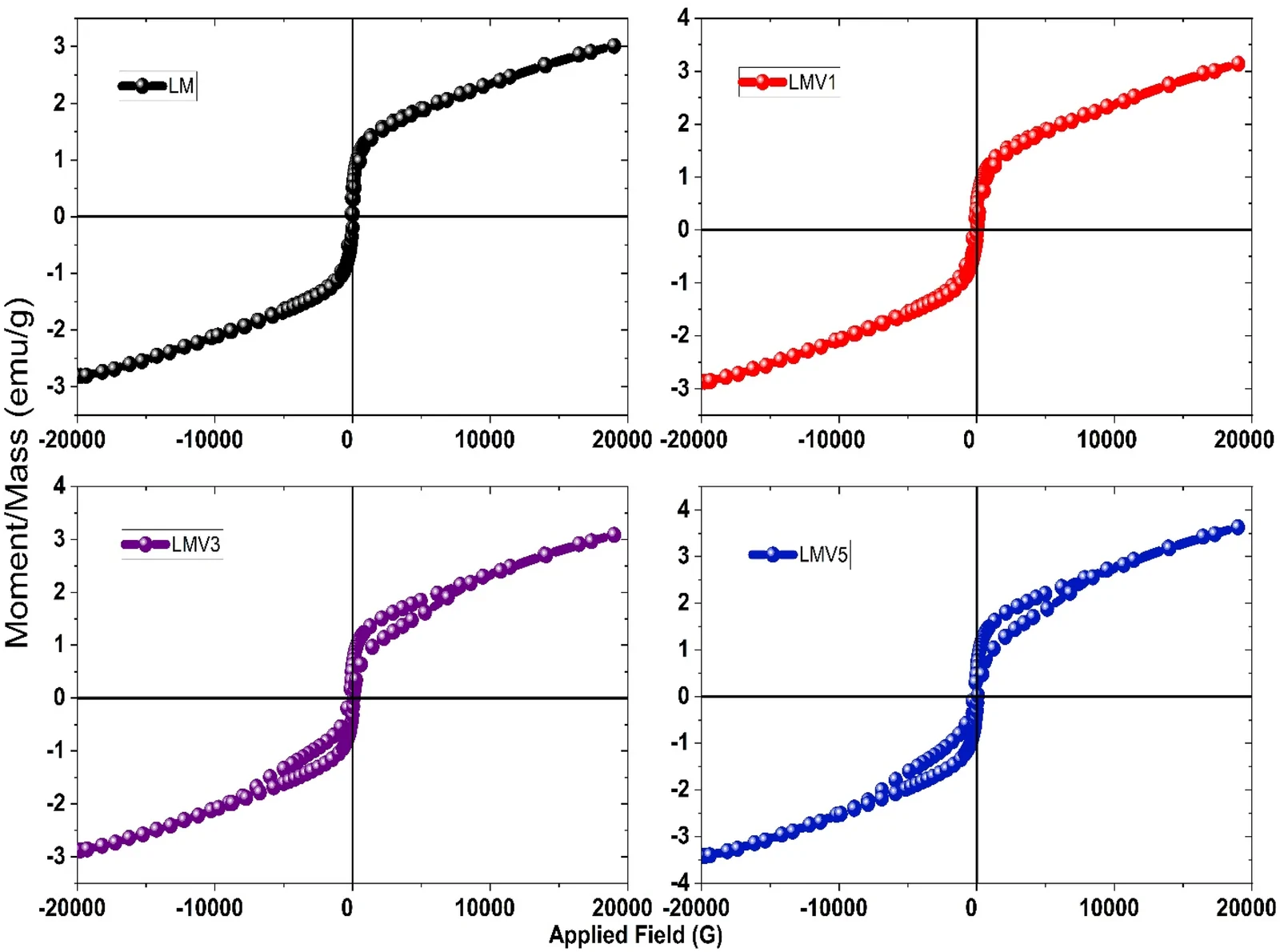
Room-temperature VSM magnetic hysteresis showing the evolution of saturation magnetization, coercivity, and remanence with V-substitution, and the hysteresis-pinching observed in LMV3 and LMV5.

**Table 3:** Room-temperature magnetic parameters of LM, LMV1, LMV3 and LMV5 derived from VSM hysteresis loops: total saturation magnetization (*M*_s(total)_), ferromagnetic saturation magnetization corrected for the paramagnetic contribution (*M*_s(FM)corrected)_) *via M*_s(FM)_ = *M*_s(total)_ − *χH*, coercivity (*H*_c_), remanent magnetization (*M*_r_), and squareness ratio (*M*_r_/*M*_s_)

Sample	*M*_s(total)_ (emu g^−1^)	*M*_s(FM)corrected_ (emu g^−1^)	*H*_c_ *G*	*M*_r_ (emu g^−1^)	*M*_r_/*M*_s_
LMO	2.91	1.67	72.0	0.318	0.109
LMV1	3.00	1.63	120.6	0.290	0.097
LMV3	2.98	1.60	173.7	0.457	0.153
LMV5	3.52	1.89	160.0	0.424	0.120

This structure-magnetism coupling is well documented in related substitution systems of LaMnO_3_. As the substitution increases, Ag-doped LaMnO_3_ exhibits an orthorhombic-to-rhombohedral transition with the magnetic moment monotonically increasing, which is attributed to the enhanced Mn^3+^/Mn^4+^ double exchange.^69^ Nanoparticles of Sr-substituted La_1−*x*_Sr_*x*_MnO_3_ further confirm that *M*_s_ and K1 in this class of materials scale with the Mn^4+^ : Mn^3+^ ratio, with a maximum at the heavily-doped, percolated-ferromagnetic composition *x* = 0.3.^70^ For the much lower levels of substitution used here (1–5%), the system is instead in the dilute-doping regime, where isolated ferromagnetic polarons/clusters nucleate around individual Mn^3+^ : Mn^4+^ pairs well below the long-range percolation threshold, the regime associated with Griffiths-phase/cluster-glass behavior rather than bulk ferromagnetism.^71^ Similar short range ferromagnetic clustering has also been observed in non-stoichiometric LaMnO_3+*δ*_.^65,72^ Both mechanisms are consistent with the cluster-glass/Griffiths-phase-like response where the blocked ferromagnetic clusters show a hysteretic behavior even above the nominal ordering temperature. In order to resolve these two possibilities, as well as determine whether there is any kind of long-range ordering below room temperature, temperature dependent magnetometry is required, but unfortunately not available for this particular work. From a different aspect, the magnetic response cannot arise from the secondary phase identified using XRD (V_2_O_5_, La_2_O_3_; Section 3.1), as both V^5+^ (*d*_0_) and La^3+^ (closed shell) are diamagnetic. This suggests the room temperature ferromagnetic-like response is likely intrinsic to double-exchange clusters and not a magnetic impurity phase.

The low coercivity reflects minimal magnetic anisotropy and easy domain reorientation, while the squareness ratio (*M*_r_/*M*_s_ = 0.109) confirms soft magnetic character typical of weakly interacting or antiferromagnetically coupled systems. Upon V-substitution, the measured saturation magnetization *M*_s_ at maximum applied field exhibits a non-monotonic trend from (2.91 emu g^−1^) in LMO to 3.00, 2.98, and 3.52 emu g^−1^ in LMV1, LMV3 and LMV5 respectively. The modest increases at 1–3% V-substitution, followed by dramatic enhancement at 5%, initially suggest progressive strengthening of magnetic interactions with substitution level. The increase *M*_s(total)_ (21% increasing) in LMV5 indicates that 5% V-substitution significantly enhances ferro-/ferrimagnetic ordering, potentially through increased density of Mn^3+^–O^2−^–Mn^4+^ pairs that enable coupling of double-exchange ferromagnetic.^73,74^

To definitively address the ferromagnetic order, Arrott plots (M^2^*vs.* H/M) were established from the room-temperature magnetization data (Fig. 11). Clear positive M^2^ intercepts were exhibited by all samples which support the presence of long-range magnetic order in both pristine and V-substituted LaMnO_3_.^32,75^ However, a clear hysteresis-pinching is observed, especially in the loops of LMV3 and LMV5 (Fig. 10). There is an area of high susceptibility enclosed by a slow-rising outer envelope. Such a shape of the loop shows that the studied samples may contain at least two components, each having its own coercive field (a soft one and a harder or less coupled one), instead of a homogeneous ferromagnetic phase. This can be explained in terms of the double valence state model (Mn^2+^/Mn^3+^/Mn^4+^), where double-exchange-coupled clusters (soft component) are incorporated into, and exchange-coupled with, the antiferromagnetic background (hard/pinning component) and suggests that the net magnetic order is better described as ferrimagnetic-like (uncompensated, unequal sublattices) rather than purely ferromagnetic.

**Fig. 11:**
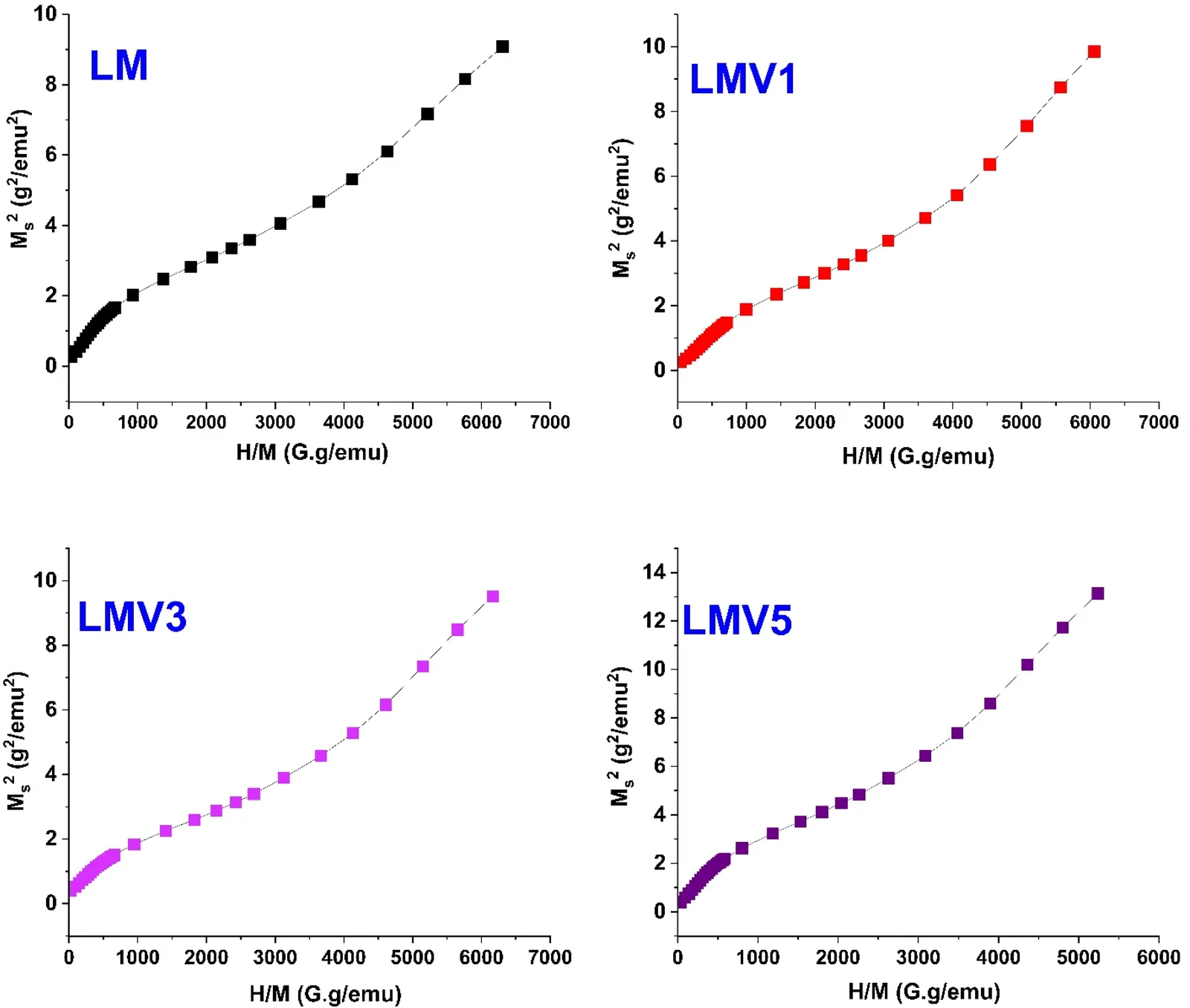
Arrott plots of pristine LaMnO_3_ (LM) and vanadium substituted sample (LMV1, LMV3 and LMV5) showing positive M^2^ intercepts consistent with long-range magnetic order in all samples.

The observed S-shaped hysteresis loops in all samples refer to unsaturated magnetization and a continued linear increase at high applied fields. This high-field linear behavior suggests that *M*_s(total)_ does not reflect pure ferromagnetic saturation but instead includes paramagnetic moments contributions. The high-field linear region of M–H curves were fitted to extract magnetic susceptibility *χ* which is followed by subtraction from total magnetization using the relation *M*_s(FM)_ = *M*_s(total)_ − *χH*.^31,76^ This correction reveals the true ferromagnetic saturation magnetization with 1.67, 1.63 1.60, and 1.89 emu g^−1^ in LMO, LMV1, LMV3 and LMV5 samples respectively. Strikingly, the ferromagnetic component represents 53–57% of the total measured magnetization in all samples which the remainder arises from paramagnetic contributions. The corrected *M*_s(FM)_ values reveal that low to moderate V-substitution (1–3%) actually decreases the intrinsic ferromagnetic moment by 2–4% compared to pristine LaMnO_3_. This is contrary to the expectations from the total magnetization data. This reduction occurs because substitution of V^5+^ introduces non-magnetic V^5+^ ions with zero magnetic moment (d^0^ electronic configuration) and creates antiferromagnetically coupled Mn^2+^ centers through charge compensation. The presence of these species results in dilution Mn^3+^–O^2−^–Mn^4+^ ferromagnetic network without contributing to ferromagnetic moment. This effectively reduces *M*_s(FM)_ despite creation of mixed-valence states. Only at 5% substitution (LMV5) does *M*_s(FM)_ increase significantly (to 1.89 emu g^−1^) with +13% increasing which indicates that the density of ferromagnetically coupled Mn^3+^/Mn^4+^ pairs finally exceeds the dilution threshold, and double-exchange interactions begin to dominate over the effects of antiferromagnetic superexchange and magnetic dilution.

The coercivity increases systematically with vanadium substitution from 72.0 G (LM), to 120.6 G (LMV1), reaching maximum at 173.7 G (LMV3), before decreasing slightly to 160.0 G (LMV5). This 2.4-fold enhancement in LMV3 represents substantial magnetic hardening compared to pristine LaMnO_3_. Critically, this occurs within confirmed long-range ferrimagnetic-like ordering, consistent with the positive Arrott-plot intercepts (Fig. 11). Such *H*_c_ enhancement could be related to the structural disorder from V-substitution that creates magnetocrystalline anisotropy: lattice expansion and Mn^3+^/V^5+^ size mismatch generates random magnetic anisotropy that impedes domain wall motion.^77^ The magnetic inhomogeneity from coexisting ferromagnetic (Mn^3+^–O^2−^–Mn^4+^) and antiferromagnetic (Mn^3+^–O^2−^–Mn^3+^, Mn^2+^-related) interactions could also create nanoscale regions where domain walls become pinned at magnetic interfaces.^36,77^ In addition, the defect pinning from charged defects (
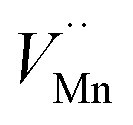
, 
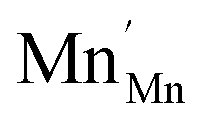
, oxygen vacancies) creates potential wells anchoring domain walls.^78,79^ The LMV5's coercivity reduction (160.0 G) despite highest *M*_s_ reveals critical microstructural effects. SEM showed extensively porous foam-like morphology with macropores and thin skeletal walls. This porous architecture contributes to *H*_c_-decreasing by weakening inter-grain exchange coupling, as air voids cause partials isolation of ferromagnetic grains. In addition, the thin magnetic walls and surface-related effects arising from the broken exchange bonds promote easier nucleation domain and magnetization reversal.^80,81^ The remanent magnetization exhibits non-monotonic evolution: LMO (0.318 emu g^−1^), LMV1 (0.290 emu g^−1^), LMV3 (0.457 emu g^−1^), LMV5 (0.424 emu g^−1^), with corresponding squareness ratios below 0.2 which confirms the soft magnetic character in all samples. LMV3 achieves the maximum stable magnetization retained after field removal of 0.457 emu g^−1^ and squareness 0.153 through optimal density of Mn^3+^–Mn^4+^ double-exchange pairs which creates robust ferromagnetic clusters and enhanced domain wall pinning from structural disorder and defects. The reduced *M*_r_/*M*_s_ ratio in LMV5 (0.120) demonstrates that microstructural connectivity is as critical as intrinsic ferromagnetic properties. This establishes LMV3 as optimal composition that balance ferromagnetic coupling, magnetic anisotropy, and structural integrity before porosity-induced degradation overwhelms the electronic benefits of higher substitution.^82^

## 4. Conclusion

Vanadium-substituted LaMnO_3_ (LMV) perovskites with substitution levels of 1–5% were successfully synthesized *via* a modified sol–gel method. Structural analysis revealed trigonal (*R*3̄*c*) structure in all samples as the major phase, together with V_2_O_5_ and La_2_O_3_ as minor phases (combined <2 wt%) in the LMV3 and LMV5 samples. Unit cell volume varies between 353.60 Å^3^ (LMO) and 355.68 Å^3^ (LMV5), with the largest change occurring between the pure and 1% samples (354.61 Å^3^), followed by smaller, saturating increases at 3% (355.53 Å^3^) and 5% (355.68 Å^3^) substitution. A crystallite size decline from 61.4 nm to 41.4 nm was achieved upon 5% of V-substitution which suggests that higher V-content suppresses growth of grains. This point is supported by the XRD indicating that the incorporation is not quantitatively complete, especially at higher nominal substitution levels (3–5%). Morphological studies revealed a development from compact grains in the LMO pristine sample to a porous, foam-like architecture in LMV5, while the compositional purity and controlled V-incorporation were verified by EDX. Optical analysis showed strong absorption across 200–1000 nm and band-gap tuning from 0.768 eV in LMO to 0.886 eV in LMV3, followed by a slight decrease to 0.841 eV upon higher V-substitution (5%). The band-gap widening at lower V levels was assigned to distortion of Mn–O–Mn and carrier depletion effects, while the red shift at higher substitution reflects increasing in the defect density. The dielectric and electrical performance of LaMnO_3_ markedly enhanced upon V-substitution. The dielectric constant reflected a 13-fold rises increase at 100 Hz which linked with the mixed Mn valence states and enhanced interfacial polarization. The conductivity also substantially increased which is attributed to polaron hopping *via* Mn^3+^/Mn^4+^ exchange. The 1% V-substituted sample exhibits maximum dielectric constant, while 3% yields the highest conductivity which indicates LaMnO_3_-tunable behavior for energy storage and charge transport applications. Impedance analysis confirmed the conduction improvement and the faster relaxation with V-substitution up to 3%, whereas higher substitution percentage (LMV5) limits charge mobility due to structural porosity. Magnetically, all samples exhibited room-temperature hysteresis indicating ferromagnetic-like component in their magnetic response. However, temperature-dependent magnetometry is required to establish the nature and ordering temperature of this component. The *M*_s_ increased from 2.91 emu g^−1^ in LMO to 3.52 emu g^−1^ in LMV5 sample while *H*_c_ rose to 174 G (LMV3) before slightly decreased at 5% due to the porous microstructure of LMV5. Vanadium substitution effectively modulates the structural, optical, dielectric, and magnetic properties of LaMnO_3_ and enables tunable functionalities for electronic, magnetic, and energy-related applications.

## Conflicts of interest

There are no conflicts to declare.

## Data Availability

The datasets used and/or analyzed during the current study are available from the corresponding author on reasonable request.
